# Relationships between dental fluorosis and fluoride concentrations in bottled water and groundwater in low-income children in Mexico

**DOI:** 10.3389/froh.2023.1187463

**Published:** 2023-06-12

**Authors:** Maria Esther Irigoyen-Camacho, Nora Perez-Perez, Marco Antonio Zepeda-Zepeda, Maria Consuelo Velazquez-Alva, Antonio Castaño-Seiquer, Ignacio Barbero-Navarro, Leonor Sanchez-Perez

**Affiliations:** ^1^ Health Care Department, Metropolitan Autonomous University-Xochimilco, Mexico City, Mexico; ^2^ School of Dentistry, Regional University of the Southeast, Oaxaca de Juárez, Mexico; ^3^ School of Dentistry, University of Seville, Seville, Spain

**Keywords:** groundwater fluoride, bottled water fluoride, fluorosis, body mass index, schoolchildren, public health, Mexico

## Abstract

**Introduction:**

The aim of the current study was to investigate associations between dental fluorosis in children living in low socioeconomic areas in Mexico, and fluoride concentrations in tap water, fluoride concentrations and in bottled water, and body mass index (BMI).

**Methods:**

A cross-sectional study involving 585 schoolchildren aged 8–12 years was conducted in communities in a southern state of Mexico with >0.7 parts per million (ppm) fluoride in the groundwater. The Thylstrup and Fejerskov index (TFI) was used to evaluate dental fluorosis, and the World Health Organization growth standards were used to calculate age-adjusted and sex-adjusted BMI Z-scores. A BMI Z-score ≤ −1 SD was used as the cut-off point for thinness, and multiple logistic regression models for dental fluorosis (TFI ≥ 4) were constructed.

**Results:**

The mean fluoride concentration in tap water was 1.39 ppm (SD 0.66), and the mean fluoride concentration in bottled water was 0.32 ppm (SD 0.23). Eighty-four children (14.39%) had a BMI Z-score ≤ −1 SD. More than half (56.1%) of the children presented with dental fluorosis in TFI categories ≥ 4. Children living in areas with higher fluoride concentrations in the tap water [odds ratio (OR) 1.57, *p* = 0.002] and bottled water (OR 3.03, *p* < .001) were more likely to have dental fluorosis in the severe categories (TFI ≥ 4). BMI Z-score was associated with the probability of dental fluorosis (TFI ≥ 4; OR 2.11, *p* < 0.001), and the effect size was 29.3%.

**Discussion:**

A low BMI Z-score was associated with a higher prevalence of dental fluorosis in the severe category. Awareness of the fluoride concentrations in bottled water may help prevent dental fluorosis, particularly in children exposed to several high fluoride content sources. Children with a low BMI may be more vulnerable to dental fluorosis.

## 1. Introduction

Fluoride is a key element in the prevention of dental caries. Fluoridated water and fluoridated salt are available to many communities across the world. These delivery methods have been successful in reducing the prevalence and severity of dental caries (1). Fluoride concentrations in drinking water should not exceed 1.5 ppm (parts per million) based on recommendations by the World Health Organization (WHO). This is intended to balance the benefits of preventing dental caries with the risks of dental fluorosis (2).

The severity of fluoride toxicity varies depending on the duration and level of exposure to fluoride (3). Chronic exposure to levels of approximately 3 ppm can cause skeletal fluorosis and an increased risk of fractures (4, 5). A longitudinal study in pregnant Mexican women identified an association between higher prenatal fluoride exposure and lower cognitive function scores in their offspring (6). In a study in Chinese children there was an association between thyroid function alterations and urinary fluoride concentration, and this association may modify the relationship between fluoride exposure and intelligence (7). Sleeping problems (8) and gastrointestinal and urinary symptoms (3) may be toxic effects of fluoride, but these links are a subject of debate and require further research to be fully understood.

The adverse effects of excessive fluoride consumption can be seen in areas where fluorosis is endemic, and such effects impact millions of people globally (9). Dental fluorosis is an irreversible condition caused by chronic high consumption of fluoride during tooth formation. Fluoride affects dental enamel and dentin during mineralization (10). Teeth with dental fluorosis exhibit demarcated lines that follow the perikamata, and the enamel looks dull white. As the fluorosis level increases, loss of dental enamel and dentin structure occurs post-eruptively and changes the appearance of the teeth. Lower enamel mineralization and increased porosity render fluorotic enamel prone to staining, and yellowish and/or dark brown areas appear on the enamel surface (11). Severe dental fluorosis negatively affects oral health and quality of life (12, 13).

Bottled water is used extensively globally, and its consumption has increased in recent years (14). In low-income countries bottled water use has a different connotation than in developed countries (15), because the tap water in underdeveloped regions may not be suitable for drinking. However, little is known about the effects of bottled water fluoride concentrations on dental fluorosis (16). Although the main cause of dental fluorosis is elevated groundwater fluoride concentrations, biological and environmental factors are also involved in the prevalence and severity of fluorosis (17). The availability of several fluoride sources also influences dental fluorosis. Environmental factors such as altitude above sea level may influence the effects of fluoride on the body, and genetic susceptibility and dietary fluoride intake have also been identified as potentially influential factors (18, 19). Nutritional status may play a role in fluoride metabolism via gastric absorption and renal excretion. These things in turn affect the bioavailability of this element, which induces changes in the tooth mineralization process and bone tissue, particularly in growing children (20).

In studies in Nigeria, India, and Mexico undernutrition has been associated with dental fluorosis (21–23). In a study in Brazilian children however, malnutrition was not related to the condition (24). Research gaps thus exist regarding the relationship between malnutrition and fluorosis (25). Poor populations in developing countries continue to face undernutrition, which is one of the main causes of death (26). In a study conducted in Mexico in 2015 one in eight children aged under 5 years were undernourished (27). Studies investigating relationships between fluorosis and undernutrition have been performed in stunted (low height-for-age) and wasted (low weight-for-height) children (21), but less severe levels of undernutrition and possible relationships between these levels and dental fluorosis have not been fully investigated.

The aim of the current study was to investigate associations between dental fluorosis in children living in low socioeconomic areas in Mexico and fluoride concentrations in tap water, fluoride concentrations in bottled water, and body mass index (BMI).

## 2. Methods

### 2.1. Study design and subject recruitment

**Figure 1:**
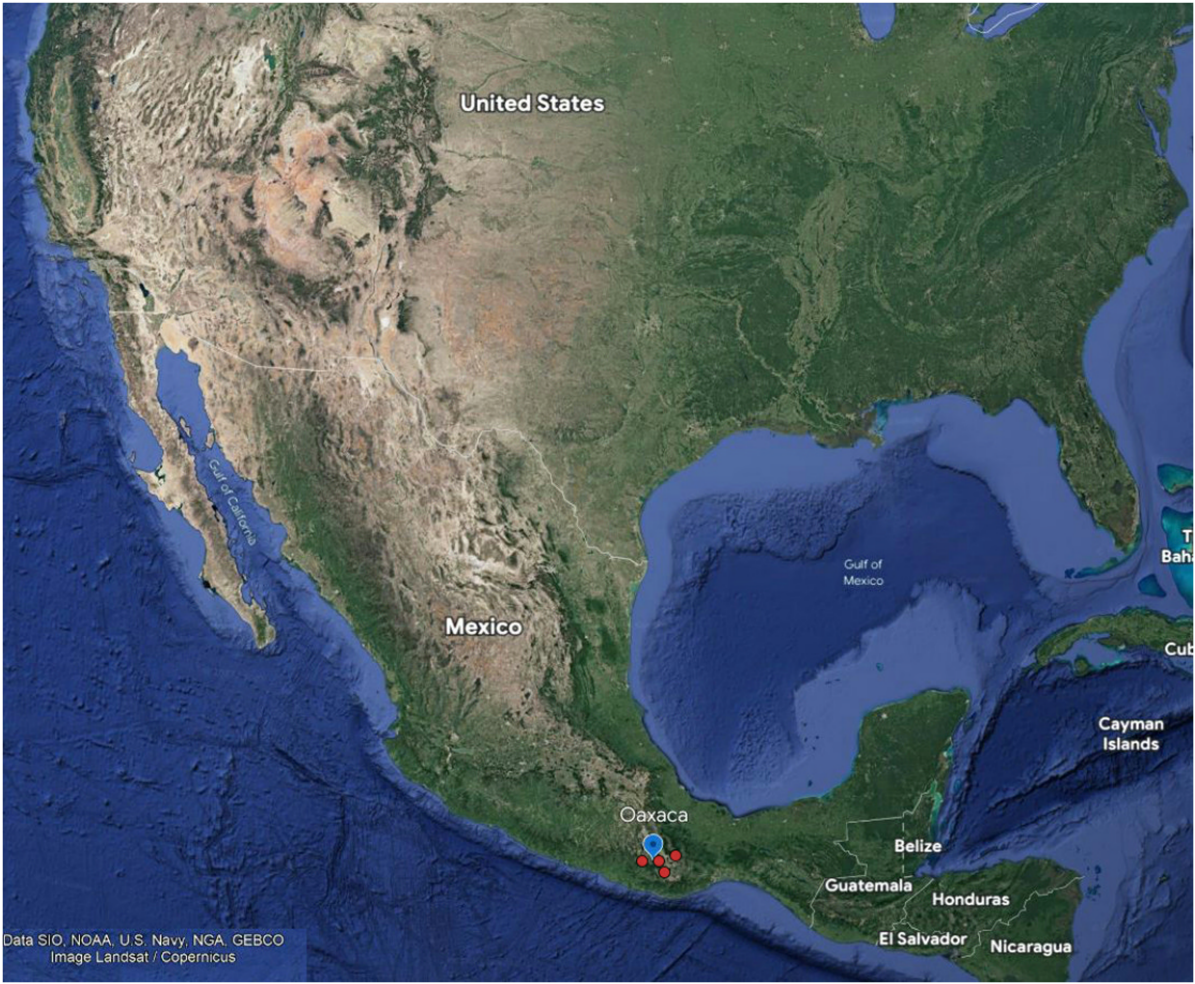
Map of Mexico, and locations of the communities studied in Oaxaca, Mex. (Modified from: Google maps Data SIO,NOAA, U.S: Navy. NGA.GEBCO. Image landsat/copernicus).

The study design was cross-sectional. The study sample was selected from the state of Oaxaca, which is in the south of Mexico. Figure 1 shows a map of Oaxaca and the districts within which the sampled communities were located. Oaxaca ranks among the four states with the highest poverty rates in Mexico, and 55% of its people live in rural areas (28). The National Population Council calculates a marginalization index to quantify poverty levels. It is a multidimensional social index that values the dimensions, forms, and intensity of exclusion in the context of development and enjoyment of benefits. Marginalization as a multidimensional and structural phenomenon is ultimately caused by the economic production model, and results in the unequal distribution of resources. The index considers four socioeconomic dimensions—education, housing, population distribution, and monetary income—and it has five levels that range from very low to very high, with the poorest localities in the very high category (29). The Oaxacan communities studied are located at the high and very high levels of the marginalization index (30).

Fluoride concentrations of water in wells in the Central and South Sierra regions of Oaxaca were used as the data frame to select communities with >0.7 ppm. The water fluoride concentrations were obtained from the National Water Commission (31) and complemented with samples obtained from water wells in the 16 communities with concentrations > 0.7 ppm, and eight were randomly selected. These communities are located between 1,367 m and 1,581 m above sea level. Details of the communities selected by district, height above sea level, and mean tap water fluoride concentrations are shown in Table 1. In these communities fluoride concentrations ranged from 0.89 to 2.67 ppm. Fluoride is naturally present in groundwater due to the weathering process. This occurs through the dissolution of fluoride-containing minerals in rocks and soils in water. These minerals include fluorspar, fluorite, apatite, sellaite, and ceramics. Volcanic activity and geothermal processes can also release fluoride into the water (32). There are several mountain ranges in Oaxaca. The South Sierra is located within the study area (Figure 1) (33).

**Table 1:** Communities selected by district, height above sea level, and mean tap water fluoride concentration.

District	Number of communities	Altitude above sea level	Mean fluoride concentration in tap water
Center	C1	1,530	1.01
Center	C2	1,530	1.68
Zaachila	Z1	1,520	0.89
Zaachila	Z2	1,570	1.36
Ejutla	E1	1,367	1.12
Miahuatlan	M1	1,581	1.68
Miahuatlan	M2	1,521	2.67
Miahuatlan	M3	1,580	2.25

The inclusion criteria were being born in the study district, not having lived outside the area for more than 6 months and being present at school during the evaluation days. The exclusion criteria were the presence of any systemic condition that required preventive medication before the oral examination, and the presence of amelogenesis or dentinogenesis imperfecta. A total of 638 students were invited to participate, of which 613 returned completed consent letters (initial participation rate 96.08%). Twenty-eight children were subsequently excluded from the study, most of whom had lived outside the area for more than 6 months or were unavailable during the examination dates. Therefore, 585 participants' data were analyzed.

### 2.2. Ethics

The study protocol was approved by the Ethics Committee of the Metropolitan Autonomous University-Xochimilco (reference no. DCBS.CD.305.18). All participating children provided signed informed consent letters in which their parents had approved their participation in the study. The assent of students' participation in the study was obtained before each oral examination was performed. Those children who needed urgent care were referred to a regional health center.

### 2.3. Oral examination

The Thylstrup and Fejerskov index (TFI) (11) was used to evaluate dental fluorosis. Children were examined while lying supine on a table under favorable natural lighting conditions using a flat mirror. Dental detritus was removed with gauze prior to dental fluorosis evaluation. The teeth were not dried before the evaluation. Two standardized examiners performed the evaluations (inter-examiner kappa 0.83, intra-examiner kappa 0.92). All permanent teeth with more than half the surface visible in the oral cavity were examined. The two teeth with the highest level of dental fluorosis were used to assess the condition at an individual level.

### 2.4. Anthropometry

A nutritionist trained in the field measured each participant's weight and height in accordance with previous guidelines based on international standards (34). The recommended anthropometric techniques were applied. The nutritionist had no information on the water fluoride concentrations of the locality being studied. Children were asked to remove their shoes and wear only light clothing for the anthropometric evaluation, for which a scale (Tanita BC-418, Japan) and a stadiometer (SECA, USA) were used. BMI was calculated using the formula kg/m^2^. Age-adjusted and sex-adjusted BMI Z-scores were obtained using the WHO Anthro Software version 3.2.2. The cut-off value for thinness was a BMI Z-score ≤ −1 SD (35).

### 2.5. Statistical analysis

Bivariate associations between dental fluorosis and the continuous variables were assessed via analysis of variance, and Pearson's chi-squared test was used for categorical variables. Associations between fluorosis status, water fluoride concentration, and nutritional status (BMI Z-score ≤ −1 SD) were determined by fitting the data to multiple logistic regression models. Fluorosis categories were dichotomized into those including enamel loss (TFI ≥ 4), and milder degrees of fluorosis. Given that the children were selected from different communities (clusters), the robust or sandwich estimator option of variance was used in the models. Marginal probabilities of response variables were estimated. The effect sizes of standardized odd ratios (ORs) were obtained from logistic regression models, and β coefficients from the model represent the percent change in log odds per one unit increase in the predictor (36). Interactions between water fluoride concentrations and BMI were tested. Model goodness of fit was determined using the Hosmer–Lemeshow test. Statistical significance was set at 0.05. The STATA version 16.1 (Stata Corp, College Station, TX) statistical software package was used for data analysis.

## 3. Results

**Figure 2:**
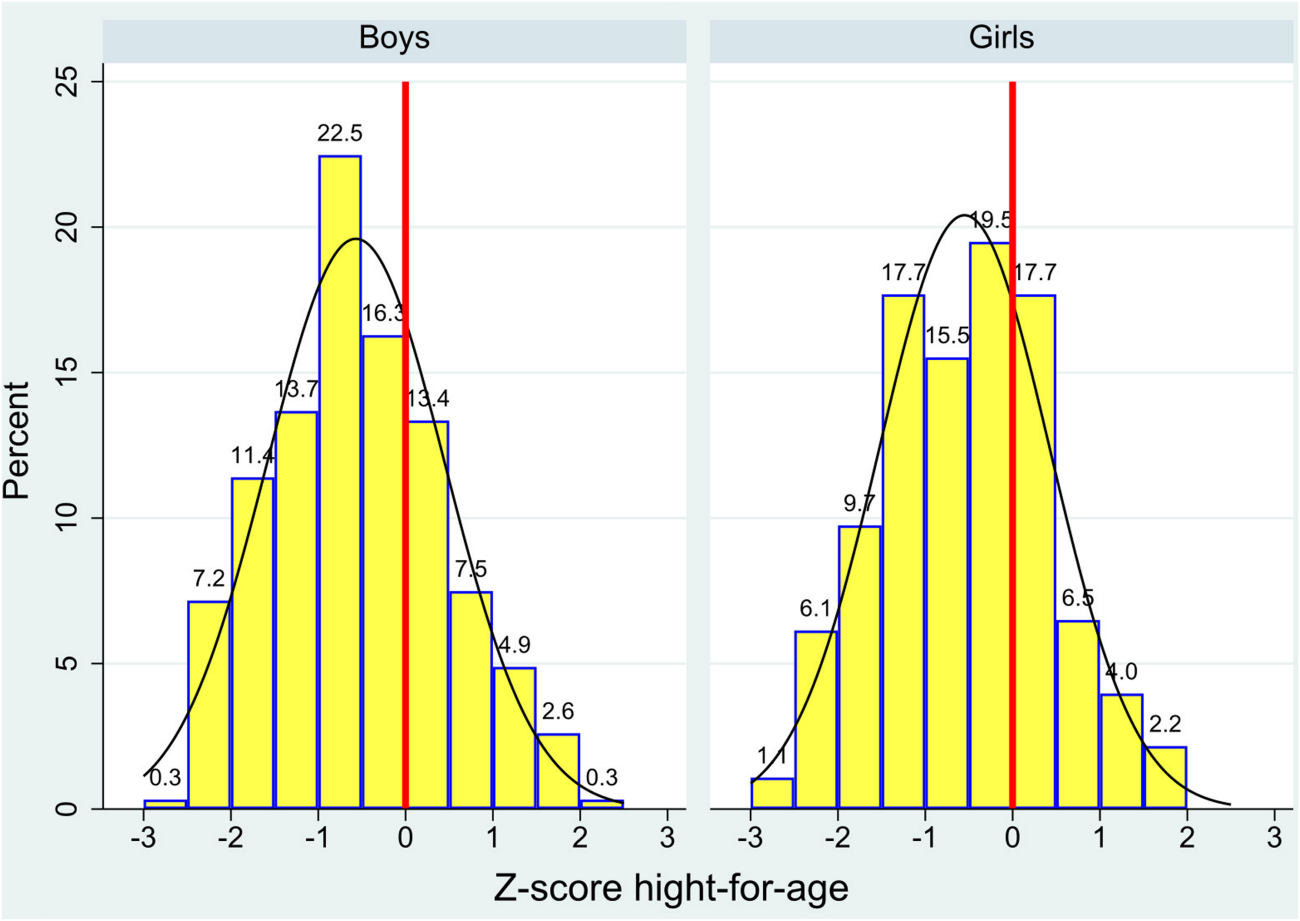
Distributions of height-for-age Z-scores in children in Oaxaca, Mexico as determined by World Health Organization reference values, by sex.

This report presents data from a total of 585 schoolchildren. Their mean age was 10.5 years (SD 0.91), and 47.35% were girls. Anthropometric characteristics of the children are shown in Table 2. The mean height Z-score was −0.63 ( SD 1.05), which is below the WHO standard population mean, and a mean BMI Z-score was 0.36 (SD 1.24) which is above the standard population mean. In girls the mean height-for-age was 0.66 (SD 0.99), and in boys it was 0.59 (SD 1.01) (*p* = 0.460). Z-score distributions for height-for-age by sex are shown in Figure 2. With respect to the WHO standards, 69.68% of boys and 71.75% of girls fell below the mean.

**Table 2:** Anthropometric characteristics of the children, main sources of drinking water, and fluoride concentrations in the drinking water in the study region of Oaxaca, Mexico (*n* = 585).

Characteristic	
Sex	*N* (%)
Girls	277 (47.35)
Boys	308 (52.65)
	mean (sd)
Age	10.5 (0.91)
Weight (kg)	36.7 (8.89)
Height (cm)	139.9 (8.41)
Height-Z-score	−0.63 (1.05)
BMI-Z-score	0.36 (1.24)
Tap water fluoride concentration (ppm)	1.39 (0.66)
Bottled water fluoride concentration (ppm)	0.32 (0.23)
	*n* (%)
**Main source drinking water**	
Bottled water	382 (65.3)
Tap water	203 (34.7)

**Figure 3:**
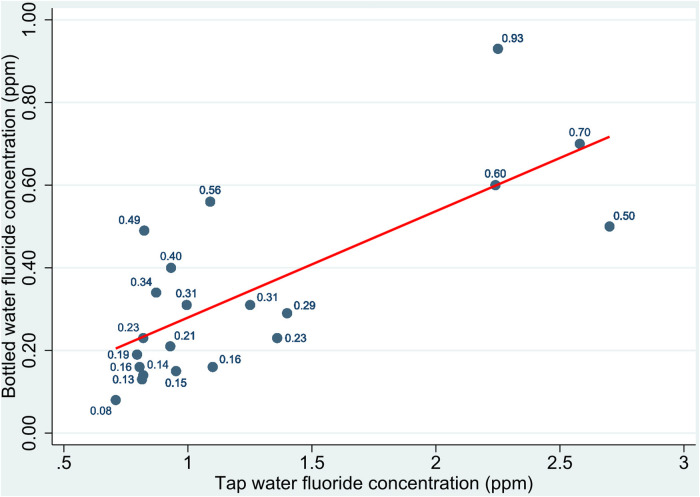
Scatter plot of distributions of fluoride concentrations in bottled water by fluoride concentrations in tap water in the communities studied in Oaxaca, Mexico.

Of 84 (14.39%) children in the study with a BMI Z-score of ≥−1 SD, 46.4% (*n* = 39) were girls and 53.57% (*n* = 45) were boys (*p* = 0.855). BMI Z-scores by age for girls and boys are shown in Table 3. The BMI Z-score in girls was 0.25 (SD 1.20), and in boys it was 0.46 (SD 1.28; *p* = 0.043). BMI Z-scores were significantly lower in 12-year-old girls than in 12-year-old boys (*p* = 0.028). No statistically significant differences were detected in other age groups. Based on the study questionnaire, 65.3% of the children consumed bottled water as their primary drinking water source, and 34.7% consumed tap water. Notwithstanding, all the children indicated that they sometimes used a combination of the two. The mean fluoride concentration of tap water was 1.39 ppm (range 0.89–2.67 ppm). The mean fluoride concentration of bottled water was 0.32 ppm (range 0.10–0.93 ppm), and 9.4% of the samples had concentrations > 0.7 ppm. A scatter plot of the fluoride concentrations of tap water and bottled water in the communities studied is shown in Figure 3. Bottled water with the highest fluoride concentrations was found in areas with higher fluoride in the water wells. Most of the children (98.6%) indicated that they brushed their teeth daily, and 89.03% of the children in this group used the same toothpaste brand, which contained 1,450 ppm fluoride.

**Table 3:** Mean body mass index Z-scores by age group in boys and girls in Oaxaca, Mexico (*n* = 585).

Age (years)	Girls	Boys	
	BMI^a^ Mean (sd) *n*	BMI^a^ Mean (sd) *n*	*p*
9	0.34 (1.08) 51	0.30 (1.41) 44	0.886
10	0.53 (1.15) 89	0.63 (1.27) 114	0.585
11	0.08 (1.29) 102	0.33 (1.26) 111	0.142
12	−0.07 (1.10) 35	0.52 (1.15) 39	0.028
9–12	0.25 (1.20) 277	0.46 (1.28) 308	0.043

^a^ BMI, body mass index (weight (kg)/height (m)^2^).

**Figure 4:**
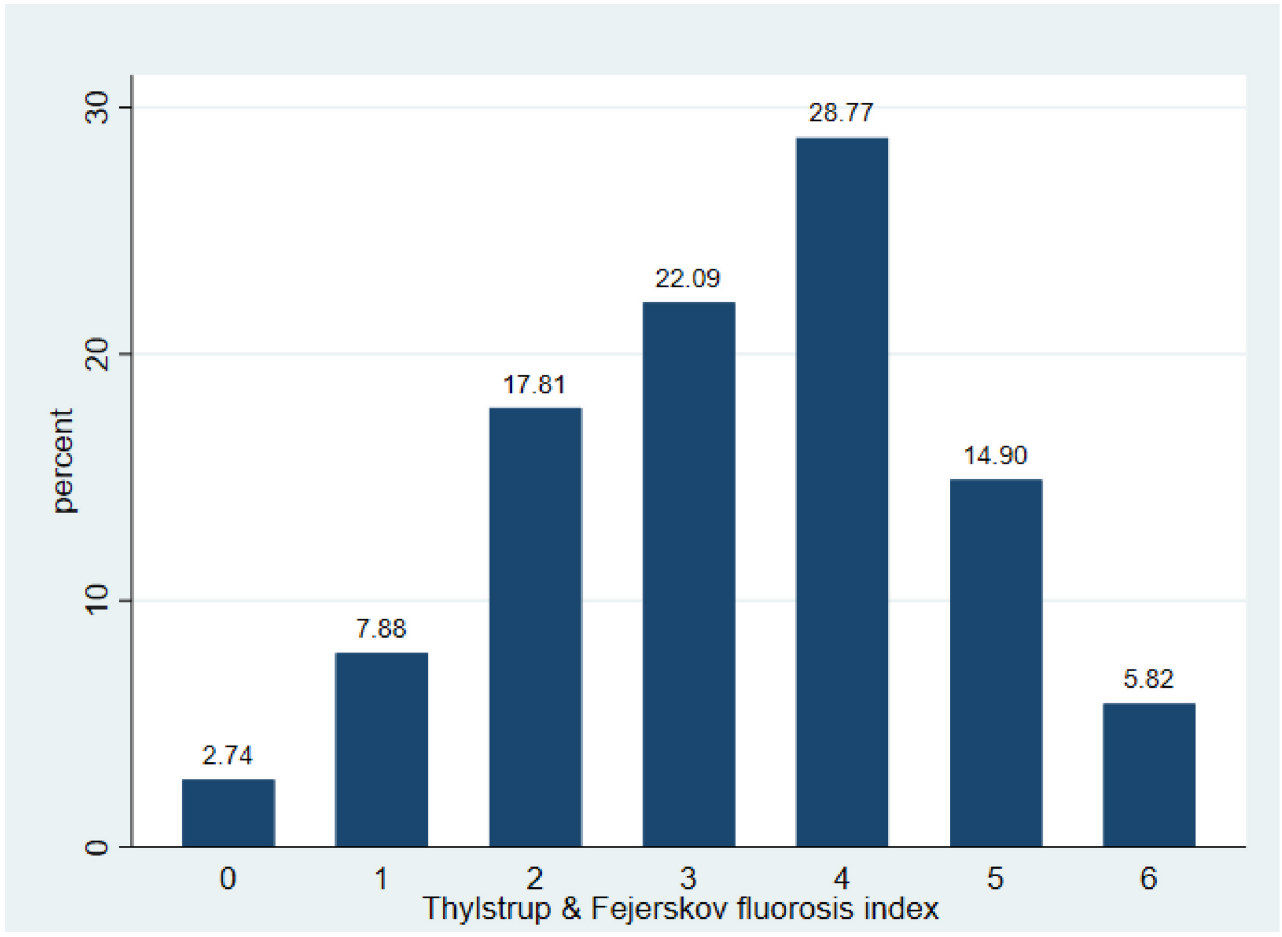
Thylstrup and Fejerskov score distribution in children in Oaxaca, Mexico.

Figure 4 depicts the TFI distribution. Less than 3% of the children had no signs of dental fluorosis, approximately one fifth had a TFI of 3, and approximately one quarter had a TFI of 4. Approximately half (49.49%) of the children had dental fluorosis in TFI categories ≥ 4. TFIs of ≥6 were found in approximately 6% of children.

Results of the logistic regression models for dental fluorosis (TFI ≥ 4) are shown in Table 4. Model 1 presents the ORs of the group of children who used tap water as their primary drinking source (*n* = 203). There was a significant association between tap water fluoride concentration and dental fluorosis (OR 2.54, *p* = 0.025), which was adjusted for age (*p* = 0.686), sex (*p* = 0.737), and BMI Z score (*p* = 0.030). Model 2 presents the results of the group of children who used bottled water as their primary drinking source. Those who used bottled water with a higher fluoride concentration (>0.7 ppm) were more likely to exhibit dental fluorosis with a TFI ≥ 4 (OR 3.39, *p* < 0.001), adjusted for age (*p* = 0.001), sex (*p* = 0.043), and BMI Z-score (*p* = 0.001). Model 3 included all children, and in that model the ORs of fluorosis in the severe categories were higher in children living in regions where tap water (OR 1.57, *p* = 0.002) or bottled water (OR 3.03, *p* < 0.001) had higher fluoride levels. Poor nutritional status, as evaluated by a low BMI Z-score, was associated with a higher likelihood of dental fluorosis (TFI ≥ 4; OR 2.11; *p* < 0.001), adjusted for age (*p* = 0.112) and sex (*p* = 0.414) (Model 3). The predicted probability of severe fluorosis in children with a low BMI Z-score was 0.69, whereas that in children with a higher BMI Z-score was 0.54. In terms of standardized OR percentage change, the effect sizes were 34.6% for tap water and 38.2% for bottled water. The change in standardized OR was 29.3% when comparing children with a low BMI Z-score (<−1 SD) with those with a higher BMI Z-score. Across the three models, a low BMI Z-score was associated with a higher likelihood of severe dental fluorosis. No significant interactions were found in the models.

**Table 4:** Crude and adjusted odds ratios for dental fluorosis and tap water fluoride concentration, bottled water fluoride concentration, and body mass index in children in Oaxaca, Mexico.

	Crude		Model 1^a^		Model 2^b^		Model 3^c^	
Crude OR	*p*	Adjusted OR^d^	*p*	Adjusted OR^d^	*p*	Adjusted OR^d^	*p*
Age	1.25 (1.02, 1.53)	0.035	1.18 (0.96, 1.29)	0.686	1.32 (1.12, 1.55)	0.001	1.18 (0.96, 1.44)	0.112
Sex	1.14	0.434	0.91	0.737	1.25	0.043	1.13	0.414
	(0.82, 1.58)		(0.51, 1.61)		(1.01, 1.55)		(0.84, 1.51)	
Tap water F concentration	1.97 (1.50, 2.58)	<0.001	2.54 (1.17, 5.48)	0.025	–	–	1.57 (1.18, 2.09)	0.002
Bottled water F^e^ concentration	5.21 (3.41, 7.96)	<0.001	–	–	3.39 (2.85, 4.04)	<0.001	3.03 (2.13, 4.31)	<0.001
BMI-z score^f^	2.32 (1.40, 3.87)	0.001	2.18 (1.11, 4.27)	0.030	2.09 (1.36, 3.22)	0.001	2.11 (1.53, 2.91)	<0.001

Goodness of fit Hosmer and Lemeshow test *p* > 0.05.

^a^ Model 1 includes only children whose main source of drinking water was tap water (*n* = 203).

^b^ Model 2 includes only children whose main source of drinking water was bottled water (*n* = 382).

^c^ Model 3 includes all children (*n* = 585).

^d^ Robust odds ratio, variance–covariance matrix of the estimators (cluster).

^e^ Baseline category F ≤ 0.7 ppm.

^f^ Baseline category BMI-Z-score ≥ −1 SD.

**Figure 5:**
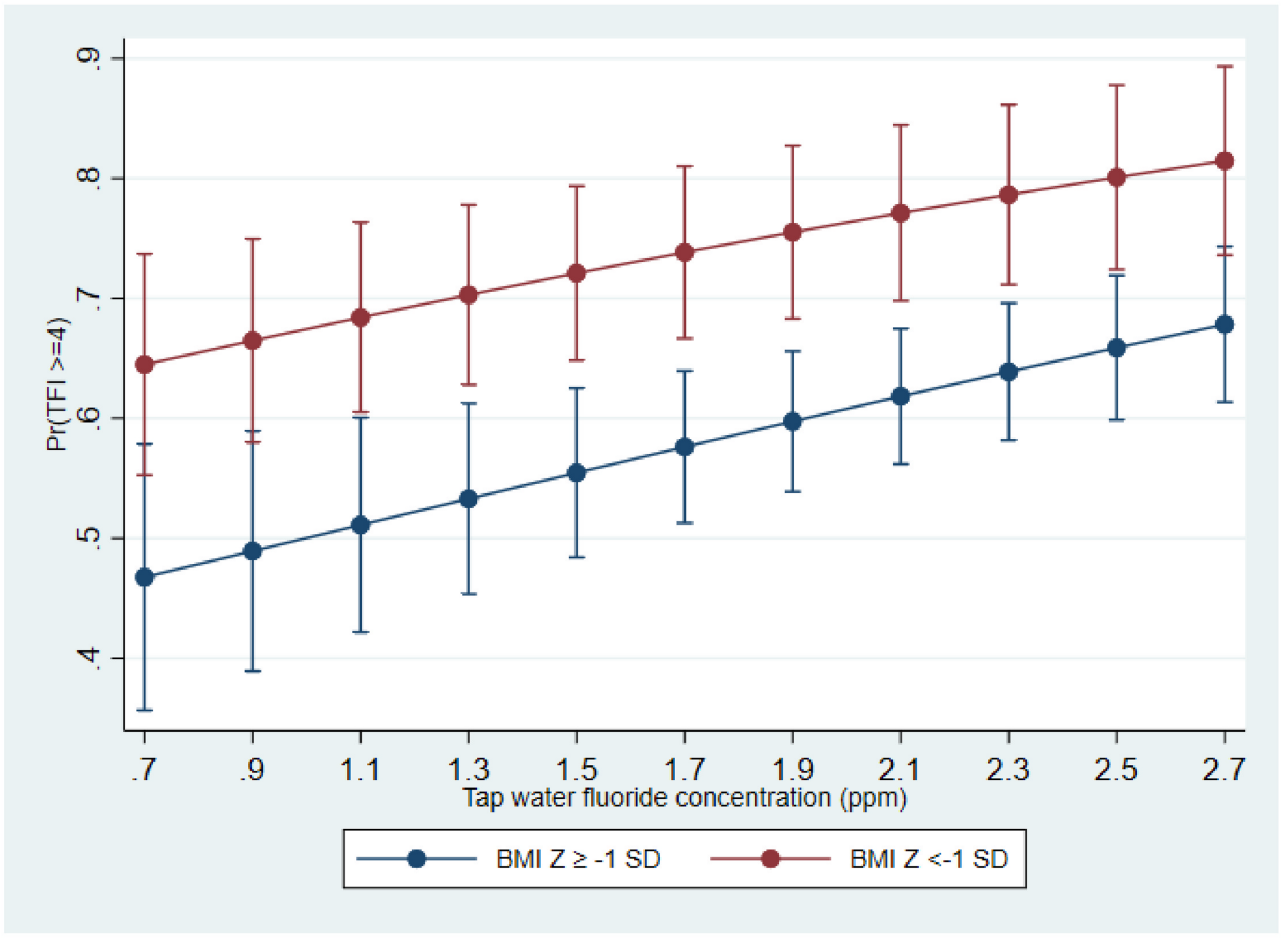
Predicted probabilities of dental fluorosis (TFI ≥ 4) and tap water fluoride concentration by body mass index Z-score category in children in Oaxaca, Mexico.

Figure 5 illustrates the predicted probability of dental fluorosis (TFI ≥ 4) by BMI category across the range of tap water fluoride concentrations for all participating children (Model 3). The predicted probabilities of fluorosis increased as fluoride content increased. In the 0.7–0.9 ppm fluoride group the predicted probabilities were 0.65 for children with low BMI Z-scores and 0.47 for children with higher BMI Z-scores. Similarly, the probabilities for children living in areas with 2.5–2.7 ppm fluoride were 0.82 for those with low BMI Z-scores and 0.58 for those with higher BMI Z-scores.

## 4. Discussion

A high prevalence of fluorosis in severe categories was detected in children exposed to tap water fluoride concentrations of ≥1 ppm. Consistently, elevated fluoride concentrations were associated with more severe fluorosis. This is concordant with the results of previous studies (37, 38). High fluoride content in tap water can cause permanent damage to the enamel of developing teeth, resulting in hypomineralization, staining, and loss of dental structures. Natural water fluoride concentrations have been identified as one of the main causes of dental fluorosis in endemic areas (17, 39). Bottled water fluoride concentrations were also associated with dental fluorosis in the current study. Small water purification plants are common in Mexico. Tap water is treated to improve its quality, but significant levels of fluoride may still be present (40). In the present study fluoride concentrations in bottled water were higher in communities where the fluoride concentration in tap water was highest.

Insufficient attention has been paid to the role of drinking bottled water in the development of dental fluorosis. Doing so may have different health effects depending on the fluoride concentration in the water. For example, where the fluoride content of groundwater is above the safe level, drinking bottled water with a low fluoride content may be recommended (41). In contrast, bottled water with a high fluoride content may contribute to dental fluorosis. The highest bottled water fluoride concentration detected in the current study was 0.93 ppm. In Oaxaca—as in several other regions of the country—there are multiple sources of fluoride exposure, including bottled water. Children in the study region can access fluoridated salt containing 200–250 mg fluoride/kg, and most use fluoridated toothpaste (42).

The WHO recommends a maximum drinking water fluoride concentration of 1.5 ppm. In the present study all the bottled water samples conformed to this recommendation. In Mexico 19-L bottles of water do not have labels indicating the fluoride content. However, in the case of natural mineral water, Mexican law stipulates that labels must include such information. Mexican regulations allow a maximum fluoride concentration of 2.0 ppm in natural mineral water and 0.7 ppm in non-mineral water (43). Considering that 19-L water bottles do not display this information, consumers cannot exercise their right to choose a better product. Concern about a lack of labeling on bottled water is evident in the United States and other countries (44, 45). The public should also have easy access to information regarding the fluoride levels in the tap water they receive at home. Fluorosis is an irreversible condition, and its treatment is expensive. In severe cases of fluorosis, a full crown reconstruction is required, which is a time-consuming and costly procedure that is often beyond the reach of low-income people.

Children in the BMI Z-score < −1 SD group had an approximately 29% increase in the standardized OR for dental fluorosis compared to those in the BMI Z-score ≥ −1 SD group. Similarly, previous studies in Nigeria, India, and Mexico indicate that undernourished children are more likely to have dental fluorosis than well-nourished children (21, 23, 46, 47). In a study in a central region in Mexico underweight children had higher levels of urinary fluoride and dental fluorosis, and fluoride excretion was consistently higher in the children with dental fluorosis (46).

Nutritional status and thyroid and kidney function were investigated in Indian children and adolescents with high and low levels of fluoride in their drinking water in a case-control study (48). In the high-fluoride areas the participants exhibited significantly reduced glomerular filtration rates, and increases in serum creatinine, which negatively affected their health. In a study on pubertal growth, fluoride exposure was associated with a significant delay in pubertal development in boys, but not in girls (49). Further research is needed to confirm these findings.

Several factors could contribute to the association between nutritional status and fluorosis. In undernourished children milk intake is likely to be low, which may lead to lower protein and calcium intake (50). Low calcium consumption in turn increases the bioavailability of fluoride, and this has been associated with skeletal fluorosis (51). Ekstrand and Ehrnebo (52) reported that the bioavailability of fluoride was 100% during fasting but ranged between 50% and 71% when milk, cheese, and yogurt were consumed. It is possible for fluoride to form insoluble compounds with calcium and other cations, and these compounds are difficult to absorb through the gastrointestinal tract. Animal studies suggest that the chronic consumption of high amounts of fluoride negatively effects calcium absorption via the downregulation of S100G expression (53). A study on endemic fluorosis areas in India identified calcium and protein deficiencies in children with defects related to osteopenia (51). Fluoride absorption, distribution, and excretion are affected by pH (20), and conditions of high gastric acidity favor fluoride absorption (54). It is possible that the undernourished children in Oaxaca in the present study consumed less calcium and protein and had longer fasting periods than those with higher BMIs, contributing to their higher levels of dental fluorosis.

A study involving Albino rats on a multigrain diet fortified with protein concluded that this diet mitigated the toxic effects of high fluoride exposure (55). The efficacy of supplementation with calcium-containing eggshell on fluoride urinary excretion and fluorosis symptoms in Ethiopian women was tested, and a six-fold reduction in urinary fluoride concentrations was reported (56). Supplementation with vitamins C and D, antioxidants, and proteins has been suggested for the prevention of fluorosis (55). This type of intervention is a promising option for the reduction of fluoride bioavailability in Mexican infants in endemic fluorosis areas. In contrast, a relationship between nutritional status and dental fluorosis was not identified in two previous studies (24, 57). The role of nutrition in fluoride bioavailability is complex. There is some evidence that overweight/obese children are more likely to have dental fluorosis and higher fluoride levels in their urine than children of normal weight (58, 59). The efficacy of dietary interventions for fluorosis prevention requires further research.

The mean height Z-score in the children in Oaxacan in the current study was negative, suggesting that they were of lower stature than the WHO standard population. This is consistent with the results of the Mexican National Health and Nutrition Survey, which found that 14.2% of the children aged under 5 years were of low height-for-age (60). The BMI Z-score had a positive mean; but one in eight children had a BMI Z-score lower than −1 SD, and these children were considered thin (35). Malnutrition, malnourishment, and excessive weight are all serious health concerns for Mexican children, as they are in other developing nations (61, 62).

The possible relationship between undernutrition and fluorosis and the concurrence of both conditions in poor countries is of concern. Underweight children may experience the negative effects of fluoride in doses that may be safe for well-nourished children. A review of groundwater fluoride studies in sub-Saharan Africa suggested that the people most affected by fluorosis were poor and living in rural areas, as were the Mexican children in the present study (39).

Early interventions to improve the health of children under 2 years of age is important, because after this age some of the effects of undernutrition become irreversible (63). Investment in nutritional interventions to prevent malnourishment will have positive effects on the health of children and ensure healthier adults, as well as a favorable economic effect on society (64).

One of the limitations of the current study was its cross-sectional design, which restricted us from drawing conclusions about causal associations between the variables investigated. Another limitation was that no data on the dietary consumption of fluoride were acquired. Notably however, the families in the regions studied have similar diets and usually do not have access to products known to contain a high fluoride concentration such as matcha green tea, brick tea, and fish with bones (65–67). Despite the study's limitations, a relationship between dental fluorosis and fluoride concentrations in tap water and bottled drinking water was observed. The results cannot be directly extrapolated to children living in communities with low tap water fluoride concentrations (≤7 ppm). Further research is required to investigate the effects of fluoride exposure from different sources on children.

A strength of the present study is the use of the TFI to assess dental fluorosis. That index contains more categories than Dean's index and provides detailed information on fluorosis status. Dean's index has been criticized for its lack of sensitivity, particularly in severe categories of this dental development defect (68). All permanent teeth were examined in the current study, facilitating a more accurate assessment of fluorosis prevalence and severity than examining only anterior teeth. The validity of the TFI has been demonstrated (69). It has been applied in numerous populations worldwide and has proven reliable. Another strength of the present study is its consideration of both tap water and bottled water. In recent years bottled water has become the main source of drinking water in several countries including Mexico. Thus, it should be considered when studying fluoride sources. It is also crucial to investigate fluoride's effects on disadvantaged children due to malnutrition being more prevalent in this population.

Policies on the regulation of the fluoride content of water and dental products should aim to achieve a balance between caries prevention and fluorosis risk reduction in the complex scenario of multiple sources of fluoride, particularly in societies with a high availability of cariogenic food, poor dental education, and low access to dental services. This is a task in which government agencies, dental professionals, and civil society should all be involved.

## Data Availability

The data that support the findings of this study are available upon reasonable request from the corresponding authors. Requests to access the datasets should be directed to meirigo@correo.xoc.uam.mx.
